# Lifetime incidence of low vision and blindness from glaucoma in a large public ophthalmology department: a retrospective cohort study

**DOI:** 10.1038/s41433-026-04660-5

**Published:** 2026-07-20

**Authors:** Amy Yen-Chi Chen, Sally Park, Anna Prasitdamrong, Peiyun Wang, Daniel Narayan, Joshua Read, Aldric Khoo, Helen V. Danesh-Meyer, Jay J. Meyer

**Affiliations:** 1 Department of Ophthalmology, Greenlane Clinical Centre, Te Toka Tumai Auckland, Auckland, New Zealand; 2 Department of Ophthalmology, New Zealand National Eye Centre, Faculty of Medical and Health Sciences, University of Auckland, Auckland, New Zealand; 3 Medical and Health Sciences, University of Otago, Dunedin, New Zealand; 4 Department of Optometry, New Zealand National Eye Centre, Faculty of Medical and Health Sciences, University of Auckland, Auckland, New Zealand

**Keywords:** Glaucoma, Vision disorders

## Introduction

Glaucoma is the leading cause of irreversible blindness worldwide [1]. Although treatment can slow disease progression, glaucoma continues to account for a substantial proportion of global blindness due to its often-asymptomatic onset and the absence of curative therapy. Despite this burden, few studies have quantified the lifetime incidence of glaucoma-related blindness and visual impairment and the most recent was conducted more than a decade ago [2]. This study examines the real-world incidence of blindness and low vision at the largest public eye centre in New Zealand.

## Methods

This retrospective cohort study was approved by the Regional Ethics Committee (#23289) and conducted in accordance with the Declaration of Helsinki. Patients were identified through the Auckland District Health Board electronic medical record system between 1 January 2016 and 31 December 2020. Clinical records were manually reviewed and two independent reviewers extracted relevant data. Any discrepancies were resolved by consulting a glaucoma ophthalmologist.

Patients were eligible if they had a confirmed diagnosis of a glaucoma subtype, defined by one of the following criteria:

1. Repeatable visual field defect consistent with glaucoma confirmed by a glaucoma consultant;
2. At least one visual field test corroborating neuro-retinal rim thinning on optical coherence tomography.
3. Visual acuity and/or visual field defect meeting World Health Organisation (WHO) Criteria [3] for blindness with advanced optic disc cupping consistent with end-stage glaucoma.

Patients with incomplete records, ocular hypertension, glaucoma suspect, or visual loss primarily attributable to another ocular or systemic condition were excluded. The primary outcome was the incidence of WHO-defined low vision or blindness attributable to glaucoma [3]. Secondary outcomes included risk factors for blindness and low vision in the primary open-angle and normal-tension glaucoma (POAG/NTG) subgroup. Statistical analyses were performed in R (version 4.4.1) by a statistician. Univariate and multivariate analyses assessed associations between clinical and demographic variables and visual impairment in the POAG/NTG cohort.

## Results

**Fig. 1:**
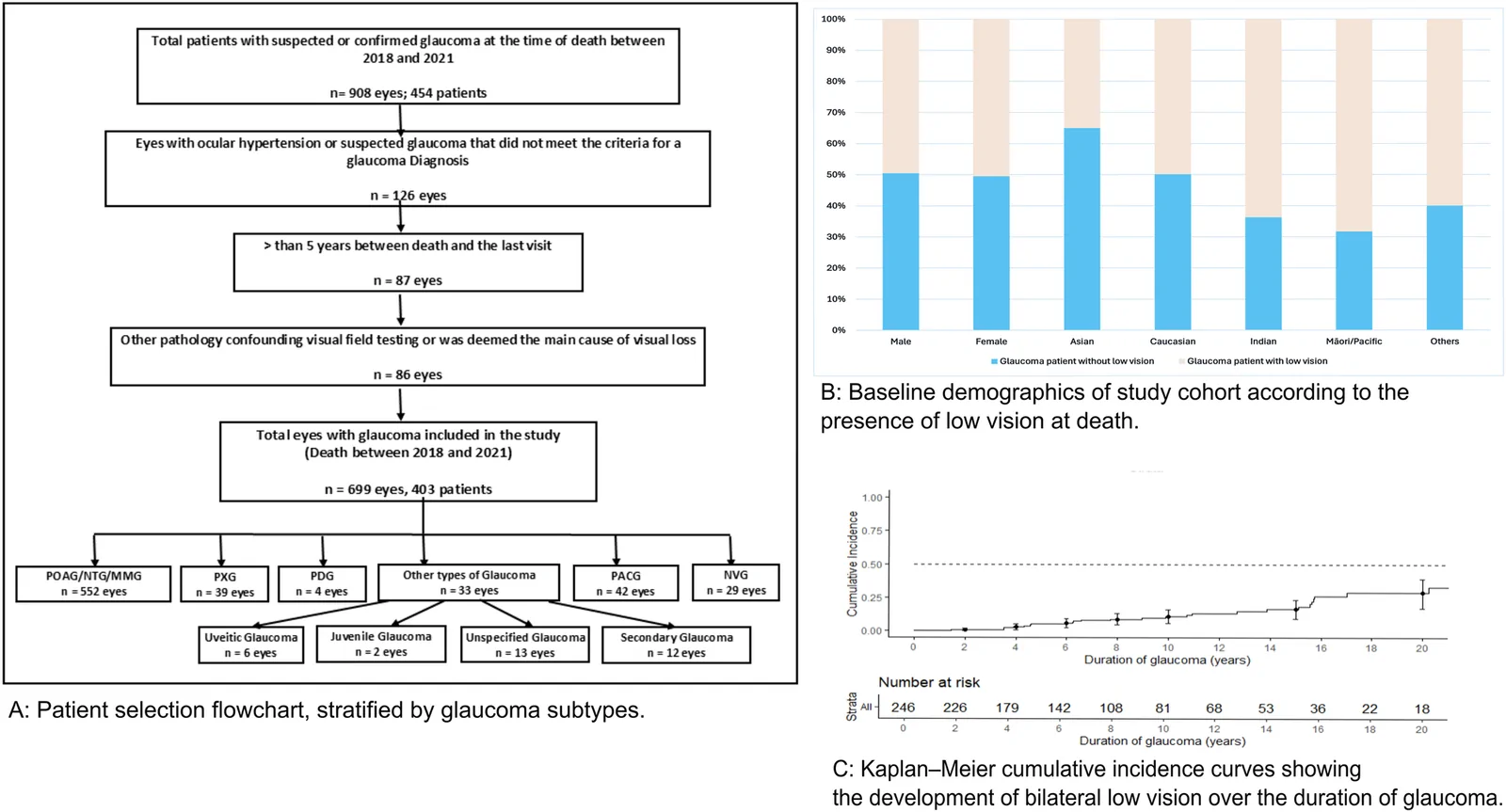
Patient selection, baseline demographics, and cumulative incidence of low vision or blindness attributable to glaucoma. **A** Patient selection flowchart, stratified by glaucoma subtypes. **B** Baseline demographics of study cohort according to the presence of low vision at death. **C** Kaplan-Meier cumulative incidence curve showing the development of bilateral low vision over the duration of glaucoma. POAG primary open-angle glaucoma, NTG normal-tension glaucoma, PXG pseudoexfoliative glaucoma, PDG pigment dispersion glaucoma, PACG primary angle-closure glaucoma, MMG mixed-mechanism glaucoma, NVG neovascular glaucoma.

A total of 454 charts were reviewed. 699 eyes from 403 patients (88.8%) were included in the analysis. The selection process and distribution of eyes by glaucoma subtype are shown in Fig. 1A and baseline characteristics of the overall cohort and patients with low vision or blindness are shown in Fig. 1B.

Overall, 25.3% (177/699) of eyes and 38.7% (156/403) of patients had low vision or blindness at their final visit before death. Bilateral low vision occurred in 10.2% of patients and bilateral blindness in 3.5%. Rates of low vision and blindness by glaucoma subtype are presented in Table 1A. Blindness in at least one eye increased with glaucoma duration, reaching 10.8% at 10 years; Kaplan–Meier estimates are shown in Fig. 1C.

**Table 1:** A: Characteristics of glaucoma patients with low vision or blindness. B: Univariate analysis assessing risk predictors of visual impairment. C: Multivariable analysis assessing adjusted risk predictors of visual impairment.

A		
	Low Vision at final visit	Blindness at final visit
**Total number of eyes**	83/699 (11.9%)	94/699 (13.4%)
**Total number of patients**	76/403 (18.9%)	80/403 (19.9%)
**Mean age (years** ± **SD)**	84.2 ± 8.34	80.4 ± 11.42
**PACG**	3 (3.6%)	5 (5.3%)
**POAG / NTG**	64 (77.1%)	57 (60.6%)
**MMG**	4 (4.8%)	2 (2.1%)
**PXG / PDS**	5 (6.0%)	9 (9.6%)
**NVG**	1 (1.2%)	13 (13.8%)
**Non-specified**	3 (3.6%)	2 (2.1%)
**Other glaucoma types (secondary & uveitic)**	3 (3.6%)	5 (5.3%)
**Juvenile glaucoma**	—	1 (1.1%)

B			
	Open-angle glaucoma patients without low vision or blindness (*n* = 176)	Open-angle glaucoma patients with low vision or blindness (*n* = 58)	*P* values
Sex			
Male	90 (51%)	29 (50%)	0.229
Female	86 (49%)	29 (50%)	
Ethnicity			
Asian	19 (11%)	3 (5.1%)	0.100
Caucasian	157 (89%)	55 (93%)	
Glaucoma severity [12] at diagnosis			
0	2 (1.1%)	0	**5.92 × 10**^**–18**^
1	82 (47%)	4 (6.9%)	
2	56 (32%)	9 (16%)	
3	26 (15%)	18 (31%)	
4	8 (4.5%)	15 (26%)	
5	2 (1.1%)	12 (21%)	
Smoking history	35 (20%)	14 (24%)	0.188
Familial glaucoma history	33 (19%)	8 (14%)	0.152
Duration of glaucoma (years)	8.6 ± 6.8	10.3 ± 7.8	0.123
Age of death (years)	84.9 ± 7.8	89.2 ± 6.1	**3.69 × 10**^**–5**^
CCT at glaucoma diagnosis (μm)	542.4 ± 38.7	534.0 ± 45.4	0.258
Intraocular pressure at presentation (mmHg)	21.2 ± 6.6	25.9 ± 9.5	**0.0007**

C			
Categories	Variables	Adjusted odds ratio (95% CI)	p-value
Demographic	Age of death	1.16 (1.04–1.31)	**0.013**
	Duration of glaucoma	1.07 (0.98–1.17)	0.145
	Gender	0.44 (0.12–1.44)	0.185
	Ethnicity	0.82 (0.09–19.61)	0.877
	Diabetes	0.98 (0.22–4.00)	0.975
	Smoking history	2.22 (0.49–9.76)	0.288
	Familial history of glaucoma	0.82 (0.21–3.00)	0.768
Clinical	Glaucoma severity	4.20 (2.45–8.26)	**2.40 × 10**^**–6**^
	IOP at presentation	1.14 (1.04–1.25)	**0.005**
	CCT	1.01 (0.99–1.02)	0.333

This table summarises:

(1) the total number of eyes and patients affected by low vision or blindness.

(2) prevalence stratified by glaucoma subtypes.

(3) mean age to develop low vision or blindness.

*POAG* primary open-angle glaucoma, *NTG* normal-tension glaucoma, *PXG* pseudoexfoliative glaucoma, *PDG* pigment dispersion glaucoma, *PACG* primary angle-closure glaucoma, *MMG* mixed-mechanism glaucoma, *NVG* neovascular glaucoma.

Categorical values chi square, *glaucoma severity* Cochran Armitage Test, numerical values = t test. Significant values are bolded. *IOP* intraocular pressure, *CCT* central corneal thickness.

Adjusted odds ratios (AORs) and 95% confidence intervals (CIs) are shown for each variable. Demographic variables included duration of glaucoma, sex, ethnicity, diabetes, smoking history and family history of glaucoma. Clinical variables included glaucoma severity, intraocular pressure (IOP) at presentation and central corneal thickness (CCT). The significant *p* values are shown in bold.

Multivariate analysis showed that greater glaucoma severity at presentation (OR 4.2, *p* < 0.001), higher intraocular pressure at presentation (OR 1.14, *p* = 0.005) and older age at death (OR 1.16, *p* = 0.013) were associated with low vision and blindness. (Tables 1B, C).

This large population study estimates the burden of glaucoma-related visual impairment. Identified risk factors emphasise the importance of earlier detection and treatment.

## Summary

### What is known about this topic

- Glaucoma remains one of the leading causes of irreversible visual loss worldwide, despite advances in glaucoma treatment.
- Real-world data describing the cumulative incidence of glaucoma-related low vision and blindness are limited.

### What this study adds

- This study demonstrated a significant cumulative incidence of low vision and blindness attributable to glaucoma in a large real-world public ophthalmology department.
- Advanced disease severity at presentation was the strongest risk predictor for subsequent low vision and blindness, highlighting the importance of earlier detection and treatment.

## Data Availability

The datasets generated during and/or analysed during the current study are available from the corresponding author on reasonable request.
